# Sharing Future Concerns About Dementia: Differences in Care and Logistical Planning Concerns Across Dyad Members

**DOI:** 10.1093/geroni/igaf122.3844

**Published:** 2025-12-31

**Authors:** Amanda Piechota, Emily Mroz, Yifan Lou, Thi Vu, Dustin Gad, Joan Monin

**Affiliations:** University of Alabama, Tuscaloosa, Alabama, United States; Emory University, Atlanta, Georgia, United States; Virginia Commonwealth University, Richmond, Virginia, United States; Yale University, New Haven, Connecticut, United States; Cornell University, Ithaca, New York, United States; Yale, Yale, Connecticut, United States

## Abstract

The progressive and degenerative symptoms of dementia cause families to develop disease-related problems or worries, or “future concerns.” Adult children represent more than half of the primary caregivers to a person living with dementia (PLWD), but may have different future concerns than their parents, causing communication and planning disconnects as they become increasingly involved in care. In this study, we aimed to 1) compare the frequency of nominating concerns related to “care and logistical planning” across adult children and parents living with memory concerns or dementia, and 2) explore which sub-types of care and logistical planning concerns may be drivers of differences across dyad members. We analyzed videos of discussions about future concerns between 122 adult children and 108 parents who self-reported memory concerns and scored 12 or higher on the Mini Mental Status Examination. Participants were asked to share three future concerns with one another. Four coders established reliability (ICC = .92, kappa = .71-.83) to quantitatively content-analyze videos for future concerns. Paired samples t-test demonstrated adult children (M= .93/2.54) shared more care and logistical planning concerns than PLWDs (M=.47/2.17, p<.001). Results were driven by adult children’s (M=.37/2.54), as compared to parents’ (M=.10/2.17) frequency of sharing concerns related to planning for PLWDs’ safety (p<.001), and adult children’s (M=.25/2.54) as compared to parents’ (M=.02/2.17) frequency of sharing concerns related to challenges with disease co-management (p<.001). Results inform development of resources to help parent- adult child dyads understand each other’s concerns, make decisions, and plan for the future.

